# Effect of the e-flipped learning approach on the knowledge, attitudes, and perceived behaviour of medical educators

**DOI:** 10.1186/s13104-022-06119-8

**Published:** 2022-06-27

**Authors:** Laleh Kian, Nahid Zarifsanaiey, Zahra karimian

**Affiliations:** E-Learning Department, Virtual School, Shiraz University of Medical Sciences, Shiraz, Iran

**Keywords:** Knowledge, Attitude, Perceived behaviour, Reaction, e-flipped learning, Educational intervention, Kirkpatrick evaluation model

## Abstract

**Objectives:**

Using the Kirkpatrick evaluation model, investigate the effect of the e-flipped learning approach on the knowledge, attitudes, and perceived behaviour of medical educators.

**Results:**

This interventional study was conducted on 140 eligible medical educators at Shiraz University of Medical Sciences (SUMS) from 2019 to 2021. A researcher-made questionnaire was developed to assess the impact of the course on three levels of reaction, knowledge, and perceived behaviour using the Kirkpatrick evaluation model. According to the findings, the average reaction and knowledge scores are higher than the desired level, but the perceived behaviour score is slightly lower than the desired level. The e-flipped learning approach can improve medical educators’ knowledge, attitude, and behaviour regarding online teaching.

**Supplementary Information:**

The online version contains supplementary material available at 10.1186/s13104-022-06119-8.

## Introduction

The widespread prevalence of COVID-19 pandemic disease has challenged educational environments. In this regard, universities use new technologies to provide their students with much more accessibility [1]. Furthermore, e-learning has become the mainstream of education in times of crisis. As the demand for online education increases, the need to improve the quality of online teaching is felt [2]. Providing quality educational programs to medical educators will be possible when educators are familiar with new technologies and apply appropriate online learning strategies [3]. Evidence shows that active and interactive learning methods, like flipped learning, help educators improve their teaching skills and use the theoretical knowledge they've learned in the real world [4].

During the coronavirus outbreak, flipped learning combined with online teaching generated a new learning paradigm known as the “e-flipped learning model” that promotes the online approach’s efficacy [5]. This strategy combines asynchronous and synchronous learning, including pre-class, in-class, and post-class learning activities. Before attending live classes, students are exposed to the material through asynchronous multimedia content. Synchronous in-class activities are typically facilitated discussions to enhance the students’ cognitive abilities. Finally, the performance of students is evaluated following the live classes. In the e-flipped paradigm, these phases should be carefully put together to get the intended learning results [5].

In addition, the quality of educational intervention should be evaluated according to appropriate standards to enable their continuous improvement [6]. Evaluating these courses and their outcomes requires an applied evaluation technique to assess the level of participants’ knowledge, attitude, and skills acquired after the course [7].

The Kirkpatrick evaluation model assesses educational programs: reaction, learning, behaviour, and outcome [8]. Reaction (learner response to the training experience), learning (following education, how much the participants learned about the subject matter). This level is typically assessed using a pretest and posttest, behaviour (whether the learning was applied in the workplace), and four outcomes (the long-term impact of training). The first three levels of the Kirkpatrick evaluation model have been assessed in some form in most academic programs. The fourth level looks at the program's long-term effects on society and requires long-term follow-up in the community [9–12].

Most medical educators provided online education throughout the COVID-19 period so that an e-flipped learning approach could improve the quality of their teaching [5]. In addition, no research has been done on educating medical educators through the e-flipped learning approach at SUMS, and an information gap is felt in this field. As a result, the present study was conducted to investigate the effect of the e-flipped learning approach on the knowledge, attitudes, and perceived behaviour of medical educators in online education.

## Main text

### Method

The present study is a one-group interventional study.

### Participants

The inclusion criteria were all educators affiliated with SUMS who enrolled in the educational intervention based on the e-flipped learning approach, with a minimum education level of a doctorate, willingness to participate, and an informed consent form. All those unwilling to continue their cooperation received similar courses, and those with extended leave of absence during the study were excluded.

### Educational intervention

This intervention included developing educational content, selecting a strategy, and implementing the intervention as follows:

#### Intervention

This e- flipped educational intervention targets medical educators involved in online teaching.

#### Learning objectives

To increase medical educators’ knowledge, attitude, and perceived behaviour about online teaching.

#### Theory

This intervention is based on constructivism, which holds that the learner constructs knowledge by doing and interacting with others [11], self-directed learning, which emphasizes students’ responsibility for their education [7], and multimedia learning to promote active and interactive learning through a variety of media such as text, audio, images, animations or videos [8]. Therefore, designing learning environments based on these theories results in an interactive, active, and flexible endeavor considered in our research.

#### Materials

Twenty multimedia content (e.g., brief videos, podcasts, power points) focused on the following topics: concepts and principles of e-learning, how to become an e-teacher, designing technology-enabled learning, online assessment methods and tools, and innovative technology in medical education.

#### Learning strategies

This course was conducted in the following manner by the e-flipped learning method [5]:

First, the participants were assigned the Learning Management System (LMS), read the course content, and completed the pretest, and then the educational materials were opened for them. Before joining the live sessions, participants read asynchronous content or performed learning activities such as dialogue with peers on a group discussion board. The Learners were guided step-by-step using icons embedded in the learning path. Eight three-hour online sessions were held over 8 weeks. First, the instructors provided learning goals, short presentations, and open-ended questions. Participants had small group discussions in eight virtual breakout rooms. The instructors engaged as facilitators, guiding and encouraging individuals to participate. The lecturers reinforced the examples using PowerPoint and educational videos and then summarized the topics.

#### Interaction

A forum for asynchronous discussion and a virtual classroom for synchronous interaction was used. Also, a social network was designed as a second method to answer the participants' questions. It involved using a second platform, such as social media, to allow interaction and engagement. The participants were also encouraged to share their experiences and additional resources on the WhatsApp group.

#### Instructors

Five e-learning professors and two physicians with 15 years of work experience in this field were responsible for teaching the course. Also, four information technology experts and two content development experts were active as technical and educational support.

#### Delivery

These courses were held through LMS over 8 weeks, including 20 asynchronous and eight online synchronous sessions.

### Data collection tools

The faculty members' knowledge, attitudes, satisfaction, and perceived behaviours about online education were evaluated at baseline and 1 week after finishing the training sessions. A researcher-made questionnaire was used based on the first to third levels of the Kirkpatrick evaluation model (additional file 1). The questionnaire consists of five parts:

1-Demographic information (age, sex, education, employment status, job duration).

Level 1. Reaction: assessing the participants’ attitude and satisfaction towards online teaching and the training course at the end of the program (41 questions).

Level 2: Knowledge: evaluating knowledge level before and after the course (12 questions).

Level 3: Perceived behaviour: evaluating the participants' performance after the course (14 questions).

The questions were designed based on the five-point Likert scale. In each dimension of the questionnaire, Scores higher than 60% were considered desirable.

### Validity and reliability

Ten experts on medical education, e-learning, and educational management were invited to comment on the questionnaire's aesthetics and content validity. The questionnaire's content validity index (CVI) was calculated on the attitude (0.86), the satisfaction (0.95), the knowledge (0.96), and the behavior (0.96) dimensions. Cronbach's alpha confirmed the reliability of the questionnaire on the attitude (0.90), satisfaction (0.94), knowledge (0.95), and perceived behaviour (0.93) dimensions.

### Statistical methods

SPSS 21 was used to analyze the collected data, including descriptive and analytical statistical tests. A paired t-test was used for comparing pre and posttest knowledge scores. Also, ANOVA and an independent-sample t-test were used to compare the differences across the socio-demographic groups. The Pearson correlation coefficient was used to examine the relationship between the three levels of the questionnaires. A P-value of 0.05 was considered the significance level.

### Sample size

One hundred and eighty faculty members of the Shiraz University of Medical Sciences participating Sciences who enrolled in the e-flipped course were selected by census method from July 2019 to December 2021. Each course lasted 8 weeks and had 20–22 participants.

## Results

Of the 180 eligible participants who started the study, 140 (79%) professors completed it. Of those, 67.9% were women, 32.1% were men, 70% were assistant professors, and 66.4% had less than 10 years of work experience. Table 1 shows a comparison of pre-and post-intervention knowledge subscale scores.

**Table 1:** Comparison of pre- and posttest knowledge subscale-scores before and after the intervention

Knowledge subscale	Before the intervention	After the intervention	Paired t-test results
	Mean $\pm$ S.D	Mean $\pm$ S.D	
Online learning concepts, theories, and principles	5.93 $\pm$ 2.86	11.43 $\pm$ 2.25	t = 22.927 p-value = 0.0004
The role of online educators	5.85 $\pm$ 3.07	11.15 $\pm$ 2.70	t = 20.217 p-value = 0.0003
Designing an online interactive learning environment	1.87 $\pm$ 1.05	3.71 $\pm$ 0.91	t = 22.163 p-value = 0.0001
Online assessment	1.96 $\pm$ 1.28	3.53 $\pm$ 1.07	t = 13.693 p-value = 0.0001
Online instructional design	1.84 $\pm$ 1.07	3.43 $\pm$ 1.05	t = 17.154 p-value = 0.0003
Emerging technologies	5.33 $\pm$ 2.62	8.83 $\pm$ 3.45	t = 14.975 p-value = 0.0008
Total knowledge	22.79 $\pm$ 10.5	42.09 $\pm$ 9.59	t = 23.749 p-value = 0.0008

**Fig. 1:**
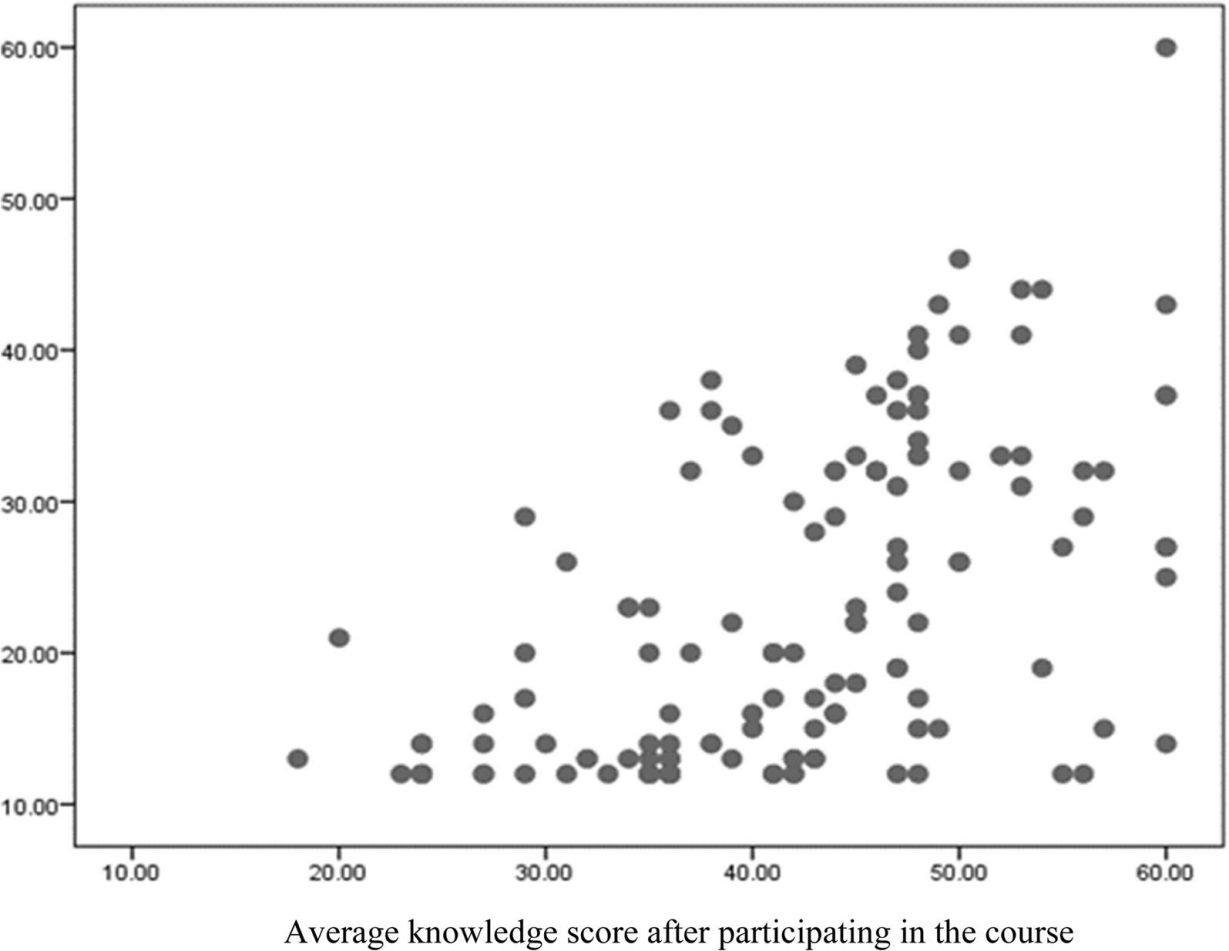
The relationship between knowledge scores before and after the intervention

The paired t-test showed that all subscales’ knowledge scores after the intervention were significantly higher. Regarding the results of the Pearson’s correlation coefficient analysis, a significant relationship was reported between the knowledge scores before and after training (p < 0.0001) (Fig. 1).

As shown in Table 2, the attitude and satisfaction mean scores are higher than the desired level (> 60%). However, the perceived behaviour score is slightly lower than the desired level. Also, the result showed that there was a significant relationship between participants' perceived behaviour, attitude, and satisfaction.

**Table 2:** Mean scores of the participants' reaction and perceived behaviour after the intervention

Variable	Number	Min	Max	Mean $\pm$ S.D
Reaction	Attitudes	2.67	5.76	4.21 $\pm$ 0.65
	Satisfaction	2.35	5	3.85 $\pm$ 0.59
Perceived Behavior		1	5	2.96 $\pm$ 0.94

The analysis of variance test (ANOVA) and an independent-sample t-test showed no significant relationship between participants' demographic variables and knowledge, attitudes, satisfaction, and perceived behaviour (Additional file 2).

## Discussion

This study aimed to assess the efficacy of e-flipped approach on the knowledge, attitudes, and perceived behaviour among medical educators in online education using Kirkpatrick’s model. According to the findings, this approach increased participants’ knowledge, attitude, and perceived behaviour towards online education. The results of the present study are consistent with the results of studies of similar teaching interventions on the level of learning and retention [12, 13], knowledge, attitude and satisfaction [14], and knowledge and skill [15] in university educators.

On the other hand, some studies show the same effectiveness for online and face-to-face educational courses [16]. Effectiveness, according to some experts, is closely connected to instructional strategies. Thus, in general, the online or face-to-face presentation method alone does not make a difference in the audience's learning [12]. In the present study, we used active and interactive learning methods, one of which was small group discussion**.** Alrehaili et al. believe that interactive learning and role-playing can capture the challenges of natural environments and enhance deep learning, intrinsic motivation, and mastery of learning [17].

Also, by providing asynchronous multimedia, the participants could study flexibly concerning time, location, and pace. Multimedia that uses the right combination of media, such as text, shape, image, video, and audio, is very appealing to learners with different learning styles, draws their attention to educational content, and improves interaction with content [18]. Furthermore, interaction with other participants was strengthened by creating group discussions synchronously through virtual breakout rooms, a forum, and a WhatsApp group. These methods support learners' interaction with classmates and teachers. Learners have different preferences for learning that can be met by using various teaching methods and improving their attitude towards educational content [3]. It also allows learners to learn with the flexibility of time and place and learning speed [19]. This way, people can apply a piece of knowledge or skill with practice and repetition. In this regard, the e-flipped approach can be used to educate medical educators.

## Limitation

The study also faced some limitations, including the following: (1) the behavioural items were self-reported responses, which could be subject to significant bias; (2) the sample came from only one university; (3) the faculty in the sample were already involved in online teaching; (4) the evaluation was a pre-post design with an evaluation that occurred 1 week after the training was completed. Given these limitations, future research should evaluate the e-flipped learning approach over a more extended period and with larger sample size.

## Supplementary Information

**Additional file1.** The questionnaire for evaluating the e-flipped learning approach on the knowledge, attitudes, and perceived behaviour of medical educators.

**Additional file2: Table S2.** The relationship between Participants' demographic and their knowledge, attitudes, satisfaction, and perceived behaviours.

## Data Availability

The data supporting this study's findings are available from the corresponding author on request.

## Abbreviations

CVI: Content validity index
SUMS: Shiraz University of Medical Sciences
LMS: Learning Management System
