# Supplementary material for: Effect of the e-flipped learning approach on the knowledge, attitudes, and perceived behaviour of medical educators
Source: BMC Res Notes. 2022 Jun 27;15:227. doi: 10.1186/s13104-022-06119-8 (PMC9235143; doi:10.1186/s13104-022-06119-8)
Supplement: Supplementary file 2 — Additional file2: Table S2. The relationship between Participants' demographic and their knowledge, attitudes, satisfaction, and perceived behaviours. [file 13104_2022_6119_MOESM2_ESM.docx]

| **Table s2. The relationship between Participants' demographic and their knowledge, attitudes, satisfaction, and perceived behaviours** | | | | | | | | | | | | | | | | | | | |
| --- | --- | --- | --- | --- | --- | --- | --- | --- | --- | --- | --- | --- | --- | --- | --- | --- | --- | --- | --- |
|  | | | **Attitude** | | | | **satisfaction** | | | | **knowledge** | | | | **perceived behaviour** | | | | |
|  |  |  | **N** | **Mean** | **Std. Deviation** | **p-value** | **N** | **Mean** | **Std. Deviation** | **p-value** | **N** | **Mean** | **Std. Deviation** | **p-value** | **N** | **Mean** | **Std. Deviation** | **p-value** |  |
| **Occupational status** | Official | | 34 | 90.2647 | 13.62775 | 0.317 | 34 | 81.8529 | 14.98511 | 0.072 | 34 | 46.3529 | 8.12360 | 0.072 | 34 | 44.0000 | 14.15927 | 0.491 |  |
|  | Conventional | | 46 | 85.6739 | 13.91730 |  | 44 | 73.6364 | 12.00018 |  | 46 | 39.5435 | 7.03233 |  | 46 | 39.7174 | 11.56943 |  |  |
|  | Contract | | 11 | 92.4545 | 13.61149 |  | 11 | 80.0909 | 15.36525 |  | 11 | 42.6364 | 13.30618 |  | 11 | 43.4545 | 12.32367 |  |  |
|  | K coefficient | | 49 | 89.0612 | 13.32668 |  | 49 | 78.2857 | 14.49856 |  | 49 | 43.6531 | 15.73948 |  | 49 | 40.8776 | 13.93980 |  |  |
| **last educational certificate** | PhD in Clinics | | 55 | 88.9455 | 13.74694 | 0.085 | 53 | 76.1887 | 13.08854 | 0.451 | 55 | 41.1455 | 10.88903 | 0.423 | 55 | 40.0909 | 10.60620 | 0.23 |  |
|  | PhD | | 78 | 87.3205 | 13.45651 |  | 78 | 78.5769 | 14.64251 |  | 78 | 44.2308 | 12.27385 |  | 78 | 41.9744 | 14.07861 |  |  |
|  | Masters and MPH | | 6 | 101.5000 | 10.11435 |  | 6 | 79.5000 | 17.28294 |  | 6 | 42.6667 | 11.20119 |  | 6 | 43.1667 | 19.10410 |  |  |
|  | Theological Sciences | | 1 | 79.0000 | . |  | 1 | 96.0000 | . |  | 1 | 34.0000 | . |  | 1 | 66.0000 | . |  |  |
| **work experience** | <5 | | 48 | 88.0417 | 14.22757 | 0.479 | 48 | 78.9167 | 14.64013 | 0.643 | 48 | 44.1458 | 16.12714 | 0.225 | 48 | 42.6250 | 14.75852 | 0.765 |  |
|  | 5-10 | | 45 | 87.3333 | 13.90389 |  | 43 | 75.7674 | 13.01983 |  | 45 | 39.6889 | 8.18486 |  | 45 | 40.9111 | 11.30129 |  |  |
|  | 10-15 | | 20 | 86.8000 | 9.86541 |  | 20 | 80.2000 | 18.30042 |  | 20 | 43.9500 | 9.40591 |  | 20 | 43.3000 | 14.74021 |  |  |
|  | 15-20 | | 8 | 95.2500 | 14.82035 |  | 8 | 73.7500 | 13.80217 |  | 8 | 42.5000 | 9.51690 |  | 8 | 39.2500 | 11.27260 |  |  |
|  | >20 | | 19 | 91.4211 | 14.56905 |  | 19 | 78.9474 | 10.65871 |  | 19 | 46.2632 | 6.47081 |  | 19 | 38.7895 | 12.26820 |  |  |
| **Sex** | male | | 45 | 87.8889 | 12.36605 | 0.71 | 45 | 76.8000 | 10.30578 | 0.55 | 45 | 43.8667 | 16.11549 | 0.49 | 45 | 38.8889 | 12.74794 | 0.11 |  |
|  | female | | 95 | 88.8000 | 14.26542 |  | 93 | 78.3226 | 15.70804 |  | 95 | 42.4105 | 8.94724 |  | 95 | 42.6737 | 13.17512 |  |  |
| **Correlations** | Age | Pearson Correlation | 1 | .060 | | | .090 | | | | -.001 | | | | -.048 | | | | |
|  |  | Sig. (2-tailed) |  | .482 | | | .294 | | | | .992 | | | | .575 | | | | |
|  |  | N | 140 | 140 | | | 138 | | | | 140 | | | | 140 | | | | |
