# Supplementary material for: Effect of the e-flipped learning approach on the knowledge, attitudes, and perceived behaviour of medical educators
Source: BMC Res Notes. 2022 Jun 27;15:227. doi: 10.1186/s13104-022-06119-8 (PMC9235143; doi:10.1186/s13104-022-06119-8)
Supplement: Supplementary file 1 — Additional file1. The questionnaire for evaluating the e-flipped learning approach on the knowledge, attitudes, and perceived behaviour of medical educators. [file 13104_2022_6119_MOESM1_ESM.doc]

**Dear Professor; Greetings and Regards**

The present questionnaire was designed to evaluate the effectiveness of the e-learning fellowship course for the faculty members of Shiraz University of Medical Sciences on the Kirk Patrick model. It is hoped that by using the results of this study, practical steps can be taken to operate and improve the quality of e-learning at Shiraz University of Medical Sciences. It should be noted that all information in this questionnaire is confidential. Thank you in advance for your time.

| **1** | Sex: □ male □ female |
| --- | --- |
| **2** | Age: ……… |
| **3** | Last educational certificate:  1.  MD 2.  PhD 3.  Masters and MPH 4.  Theological Sciences |
| **4** | Field of Study: …………………… |
| **5** | Occupational status:  1. Official 2.Conventional 3.Contract 4. K coefficient |
| **6** | Academic Rank  1. Master 2.  Associate Professor 3.  Assistant Professor 4.  Instructor |
| **7** | University of work: ………………….. |
| **8** | School of service:  1.  Medicine 2.  Dentistry 3.  Pharmacy 4.  Midwifery Nursing  5.  Paramedical 6.  Health 7.  Nutrition and Food Science 8.  Rehabilitation  9.  Management and Medical Information 10. New Sciences and Technologies  11.  Virtual College 12.  Research Centers |
| **9** | work experience   1. less than five years 2.  5- 10 years 3. 10-15 years 4.  15 -20 years   5.  more than 20 years |
| **10** | which e-learning course have you participated in?  Please bring the year and month if you remember: ……………………….. |
| **11** | What other virtual trainings do you need in your job? Please note: …………. |

**Attitude questionnaire of faculty members about virtual education and e-learning**

| Totally Opposition | Opposition | Relatively opposite | Relatively Agree | Agree | Totally Agree | Questionnaire items |  |
| --- | --- | --- | --- | --- | --- | --- | --- |
|  |  |  |  |  |  | I prefer face-to-face learning to e-learning | 1 |
|  |  |  |  |  |  | I feel comfortable in virtual communication, and I can easily convey my thoughts and feelings | 2 |
|  |  |  |  |  |  | I like to try a variety of virtual learning experiences in teaching | 3 |
|  |  |  |  |  |  | I feel that using e-learning is a waste of student and teacher time. | 4 |
|  |  |  |  |  |  | I'm concerned about intellectual property and classroom privacy in e-learning | 5 |
|  |  |  |  |  |  | I do not want to use e-learning | 6 |
|  |  |  |  |  |  | I feel that e-learning motivates students more | 7 |
|  |  |  |  |  |  | I believe that in e-learning, high-quality learning occurs | 8 |
|  |  |  |  |  |  | In my opinion, the implementation of e-learning increases the possibility of interaction, exchange and cooperation between students | 9 |
|  |  |  |  |  |  | E-learning provides an excellent interactive space between learners. | 10 |
|  |  |  |  |  |  | E-learning provides the basis for knowledge exchange and sharing. | 11 |
|  |  |  |  |  |  | Assignments in e-learning can enhance students' critical thinking. | 12 |
|  |  |  |  |  |  | In virtual education, teachers are more careful in choosing educational content | 13 |
|  |  |  |  |  |  | Virtual discussion space is essential in student learning | 14 |
|  |  |  |  |  |  | With virtual education, I can provide more effective teaching | 15 |
|  |  |  |  |  |  | Along with using virtual training, my computer skills are strengthened | 16 |
|  |  |  |  |  |  | Virtual education has no application in medical sciences | 17 |
|  |  |  |  |  |  | Traditional education does not meet all the needs of the new generation of students. | 18 |
|  |  |  |  |  |  | With the current trend, tomorrow's universities are inconceivable without the use of virtual education | 19 |
|  |  |  |  |  |  | Virtual education is effective in developing educational justice in the long run | 20 |
|  |  |  |  |  |  | I recommend using virtual training for teaching to my other colleagues | 21 |

**E-learning fellowship educational questionnaire from the perspective of faculty members participating in the course**

| Totally Agree | Agree | intermediate | Opposition | Totally Opposition | **Items** | | |
| --- | --- | --- | --- | --- | --- | --- | --- |
| **5** | **4** | **3** | **2** | **1** |  |  |  |
|  |  |  |  |  | This course encouraged me to use e-learning capabilities | **Knowledge** | **1** |
|  |  |  |  |  | The goals and content of the application were in line with my needs |  | **2** |
|  |  |  |  |  | The content was new and up to date |  | **3** |
|  |  |  |  |  | What I learned was effective in enhancing my knowledge of e-learning |  | **4** |
|  |  |  |  |  | I think my expectations of the scientific content of the material were well met |  | **5** |
|  |  |  |  |  | I was not stressed during the period, and I could easily ask my questions | **communications** | **6** |
|  |  |  |  |  | This course helped to increase self-confidence in the use of virtual education and teaching |  | **7** |
|  |  |  |  |  | There was an excellent scientific relationship between the participants and the professors |  | **8** |
|  |  |  |  |  | Participating in group discussions, forums, chats, etc. was an exciting and valuable experience |  | **9** |
|  |  |  |  |  | Visual interactions between professors and course participants were desirable and effective |  | **10** |
|  |  |  |  |  | The course instructors had sufficient knowledge and information | **Teachers** | **11** |
|  |  |  |  |  | The course instructors provided the conditions for suggestions and comments |  | **12** |
|  |  |  |  |  | Clear explanations and examples were provided to understand the content |  | **13** |
|  |  |  |  |  | Time and content management of the courses were well done |  | **14** |
|  |  |  |  |  | The moderators of this course gave good feedback to the participants |  | **15** |
|  |  |  |  |  | The educational environment was scientific and favourable | **General feeling** | **16** |
|  |  |  |  |  | Participating in this course made me feel good |  | **17** |
|  |  |  |  |  | I had no problem asking questions and ambiguities |  | **18** |
|  |  |  |  |  | I rarely got tired during this period |  | **19** |
|  |  |  |  |  | I recommend participating in this course to my other colleagues |  | **20** |

**Self-assessment questionnaire before and after the e-learning fellowship course**

| **Knowledge after the course** | | | | | | | | | | **Knowledge before the course** | | | | | **Titles** | | |
| --- | --- | --- | --- | --- | --- | --- | --- | --- | --- | --- | --- | --- | --- | --- | --- | --- | --- |
| **5** | | **4** | **3** | | **2** | | **1** | | | **5** | **4** | **3** | **2** | **1** |  |  |  |
|  | |  |  | |  | | | |  |  |  |  |  |  | e-learning in medical education | **Online learning concepts, theories, and principles** | **1** |
|  | |  |  | |  | | | |  |  |  |  |  |  | The concepts, basics, and components of e-learning |  | **2** |
|  | |  |  | |  | | | |  |  |  |  |  |  | Learning Management System (LMS) |  | **3** |
|  | |  |  | |  | | | |  |  |  |  |  |  | The role of an e-teacher | **The role of online teachers** | **4** |
|  | |  |  | |  | | | |  |  |  |  |  |  | The principles of teaching in the virtual classroom |  | **5** |
|  |  | | |  | |  | |  | |  |  |  |  |  | Preparing an electronic course plan |  | **6** |
|  |  | | |  | |  | |  | |  |  |  |  |  | Designing interactive tools and approaches in virtual education | **Designing an online interactive learning environment** | **7** |
|  |  | | |  | |  | |  | |  |  |  |  |  | Methods and approaches for students' online evaluation | **Online assessment** | **8** |
|  |  | | |  | |  | |  | |  |  |  |  |  | Designing multimedia in an e-learning environment | **Online instructional design** | **9** |
|  |  | | |  | |  | |  | |  |  |  |  |  | Mobile learning in medical education | **Emerging technologies** | **10** |
|  |  | | |  | |  | |  | |  |  |  |  |  | Virtual reality &Augmented reality |  | **11** |
|  |  | | |  | |  | |  | |  |  |  |  |  | Gamification and game-based learning |  | **12** |

**Evaluate the perceived behaviour of faculty members participating in the e-learning fellowship course**

| **never** | **rarely** | **sometimes** | **often** | **Always** | **Titles** | |
| --- | --- | --- | --- | --- | --- | --- |
|  |  |  |  |  |  |  |
|  |  |  |  |  | I use e-learning capabilities in teaching. | **1** |
|  |  |  |  |  | I hold a virtual class in the e-learning management system. | **2** |
|  |  |  |  |  | I upload instructional content and assignments to the e-learning management system. | **3** |
|  |  |  |  |  | I follow the principles of teaching when holding a virtual class. | **4** |
|  |  |  |  |  | I design electronic courses. | **5** |
|  |  |  |  |  | I use group discussions in the e-learning system to interact with students. | **6** |
|  |  |  |  |  | I use social media to educate and interact with students. | **7** |
|  |  |  |  |  | I take my exams electronically | **8** |
|  |  |  |  |  | I design my text and training scenario based on the principles and standards of multimedia in the e-learning environment. | **9** |
|  |  |  |  |  | With the help of others, I can create some of my e-learning content. | **10** |
|  |  |  |  |  | I use mobile learning technology. | **11** |
|  |  |  |  |  | I use augmented reality technology in practical classes. | **12** |
|  |  |  |  |  | I use the game-making method in e-learning. | **13** |
|  |  |  |  |  | I use logbooks and electronic portfolios to grade and evaluate students. | **14** |
